# The Usefulness and Feasibility of Mobile Interface in Tuberculosis Notification (MITUN) Voice Based System for Notification of Tuberculosis by Private Medical Practitioners – A Pilot Project

**DOI:** 10.1371/journal.pone.0138274

**Published:** 2015-09-16

**Authors:** Banurekha Velayutham, Beena Thomas, Dina Nair, Kannan Thiruvengadam, Suma Prashant, Sathyapriya Kittusami, Harivanzan Vijayakumar, Meenachi Chidambaram, Shri Vijay Bala Yogendra Shivakumar, Lavanya Jayabal, Ashok Jhunjhunwala, Soumya Swaminathan

**Affiliations:** 1 National Institute for Research in Tuberculosis (formerly Tuberculosis Research Centre), Chennai, Tamil Nadu, India; 2 Indian Institute of Technology Madras (IITM)’s Rural Technology and Business Incubator (RTBI), Chennai, Tamil Nadu, India; 3 District Tuberculosis Officer, Chennai, Tamil Nadu, India; The Foundation for Medical Research, INDIA

## Abstract

**Introduction:**

Tuberculosis (TB) is a notifiable disease and health care providers are required to notify every TB case to local authorities. We conducted a pilot study to determine the usefulness and feasibility of mobile interface in TB notification (MITUN) voice based system for notification of TB cases by private medical practitioners.

**Methodology:**

The study was conducted during September 2013 to October 2014 in three zones of Chennai, an urban setting in South India. Private clinics wherein services are provided by single private medical practitioners were approached. The steps involved in MITUN included: Registration of the practitioners and notification of TB cases by them through voice interactions. Pre and post-intervention questionnaires were administered to collect information on TB notification practices and feasibility of MITUN after an implementation period of 6 months.

**Results:**

A total of 266 private medical practitioners were approached for the study. Of them, 184 (69%) participated in the study; of whom 11 (6%) practitioners used MITUN for TB notification. Reasons for not using MITUN include lack of time, referral of patients to government facility, issues related to patient confidentiality and technical problems. Suggestions for making mobile phone based TB notification process user-friendly included reducing call duration, including only crucial questions and using missed call or SMS options.

**Conclusion:**

The performance (feasibility and usefulness) of MITUN voice based system for TB notification in the present format was sub-optimal. Perceived problems, logistical and practical issues preclude scale–up of notification of TB by private practitioners.

## Introduction

Tuberculosis (TB) has been a notifiable disease under the Public Health (Control of Disease) Act 1984 and Public Health (Infectious Diseases) Regulation of 1988 **[1,2]**. According to an order (7^th^ May, 2012) issued by the Govt. of India, Ministry of Health and Family welfare, health care providers are required to notify every TB case to local authorities **[3]**. TB patients in India are diagnosed and managed both in the public and private sector **[4,5]**. The Revised National TB Control Programme (RNTCP) has formulated a web portal to facilitate TB notification known as NIKSHAY for both public and private health care providers to minimize variations in notification practice and improve the quality of data in the local TB surveillance system **[6]**. A private practitioner can provide details of TB patients via paper or email to the Nodal Officer who will then upload it in the NIKSHAY website. This method has its inherent difficulties which includes the possibility of details not reaching the Nodal Officer when sent as hard copy or problems due to internet access. A procedure which is simple, convenient and links the private practitioner directly to the Nodal Officer of RNTCP to notify TB patients is needed.

Mobile phone technology is increasingly used in monitoring health outcomes in disease management and delivering health interventions aimed at disseminating information and bringing about a positive behaviour change **[7]**. Text based and voice based mobile data systems have been shown to be innovative and successful models in data collection **[8,9]**. These are promising tools which could be used in TB disease notification with potential to lower the burden on the private providers. Based on our request, the Indian Institute of Technology Madras (IITM)’s Rural Technology and Business Incubator (RTBI) developed a mobile phone based voice enabled TB notification system. In this method, notification is achieved by making a simple voice call through a mobile phone to a designated number and providing patient details. The information that is provided during that voice call can be viewed on a web-interface for day-to-day surveillance of TB Notification.

We conducted a pilot study to determine the usefulness and feasibility of Mobile interface in TB notification (MITUN) voice based system for notification of TB cases by private medical practitioners.

## Materials and Methods

The study was conducted during September 2013 to October 2014 in Chennai, which is a city of 7 million people in South India. Permission was obtained from the health authorities for the conduct of the study.

### Description of MITUN

TB Notification Voice-Based application uses Voice Net, a Personalized Voice based Information Retrieval and Transaction System (PVIT) jointly developed by IITM’s RTBI and its incubated company, Uniphore Software Systems. Voice Net is essentially a system that can retrieve and deliver information to end-users over the mobile phone by nothing more than voice interactions. The information is categorized into domains and into sub-domains, so that the necessary information can be obtained by walking through the set of questions, which guide the users towards the specificity of the information they are looking for.

The technology has a unique Multi-lingual Speech recognition Program for data capture and intelligent analytics tools at the backend for speech to text conversion enabling remote voice based data collection and viewing of real time data on a web portal. Unlike other voice based tools that leverage speech recognition, this system does not require training of the speech engine. Hence, the system supports several languages i.e. the voice recording and voice prompts can be changed to any particular language or dialect of the chosen target audience, enabling it to be fine-tuned to suit user-requirements and customized accordingly. The basic architecture of VoiceNet and its usability on low-end mobile phones enables the services to scale easily across states and geographies without significant investment and is independent of service provider. The speech to text conversion for non-categorical information was done by a designated staff.

**Fig 1:**
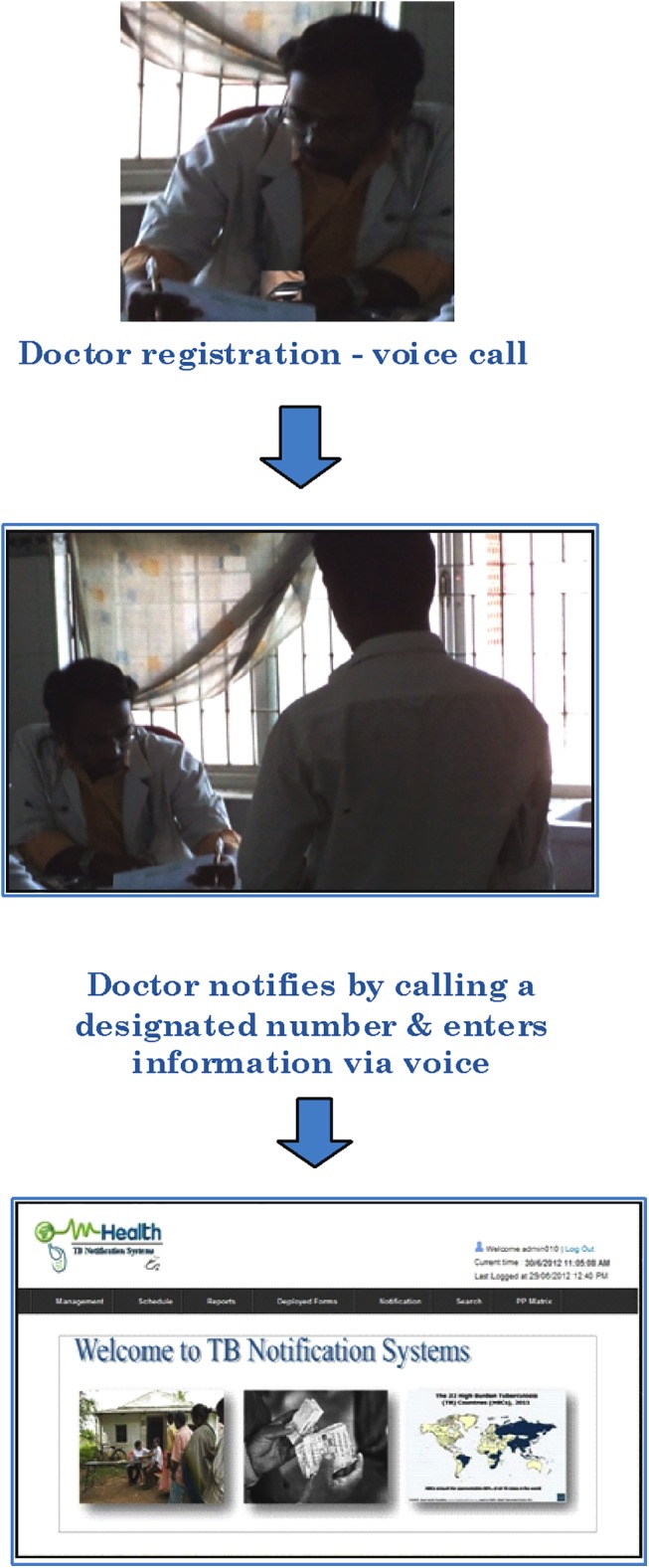
Steps involved in TB case notification by private medical practitioners using MITUN.

The process involved in TB notification through MITUN is shown in Fig 1.

#### Registration of Private Medical Practitioners

The Private Medical Practitioners were informed about the designated telephone numbers for MITUN. There were 10 telephonic lines allotted for the designated number. The following details were to be provided by the private practitioner for completing his/her one-time registration individually: Name, Medical Registration number, phone number–landline / mobile no and complete address. Registration took about 2 minutes.

#### Notification of TB cases

Diagnosed or started on treatment was carried out via mobile phone by calling the same designated MITUN telephone number and supplying patient details as required by NIKSHAY. The medical registration number is the unique identification number for the practitioners who are registered in MITUN. While using this application, the private practitioner navigates through the various options given in the system using voice prompts and enters relevant information for notification of TB cases. TB notification for each patient takes about 5 minutes. After completion he/she can either exit the system and end the call or return to the system and notify the next patient. This process can be carried out through a simple call from a mobile number. The following patient related information was requested: Name of the patient, patient’s Fathers/Husbands Name, patient’s age, gender, Govt. of India issued Identification number (Aadhar, Driving license, ration card), residential address, phone number, type of TB: Pulmonary / Extrapulmonary / Both, category of TB: New / Recurrent / Treatment change, date of TB diagnosis, investigations done, date ATT prescribed, anti-TB drugs: First line / Second line.

Sample MITUN call for TB notification is provided in Table 1.

**Table 1:** Sample Interactive Voice Response System (IVRS) call for TB notification.

	Notification Prompts
**System**	Greetings Dr.----------, you can start notifying by answering the following questions specific to patient details
**System**	Name of the patient
**Doctor**	**<Mahesh>**
**System**	Father/Husband Name
**Doctor**	**<Manikandan>**
**System**	**Age.** For example, if the patient is 37 years old, say ‘thirty seven’
**Doctor**	**<37>**
**System**	Gender
**Doctor**	**<Male>**
**System**	Say the Government of India issued identification number
**Doctor**	**<A10041PK0>**
**System**	Patient’s complete residential address
**Doctor**	**IIT Research park, Taramani, chennai**
**System**	6 Digit area pin code
**Doctor**	**<600045>**
**System**	Say the patient’s phone number one by one clearly
**Doctor**	**<4466498932>**
**System**	To start entering the patient’s TB history say whether the site of disease is “**Pulmonary” or “Extra pulmonary**
**Doctor**	**<Pulmonary>**

### Study procedures

The study procedure involved mapping of all the private medical practitioners in the 3 selected Tuberculosis Units (TUs) namely Tondiarpet, Pulianthope and Thiru Vi Ka Nagar. A Private practitioner / Clinic (single) included any Health Establishment where TB cases are treated or diagnosed clinically / radiologically and the medical services are provided by a single medical practitioner.**[6]** The practitioners were approached individually by trained study staff.

A pre-intervention questionnaire was completed by practitioners who were willing to participate in the study. Questions were related to professional details, specialization, number of TB cases diagnosed/treated in the past 6 months, awareness of Govt. order on TB notification, comfort in notifying TB cases, ever notified TB cases and the convenient modality to notify TB cases. Those practitioners who were willing to notify TB cases through MITUN were given a demo of procedures, which included one-time registration and subsequent TB notification as mentioned above.

Medical practitioners had requested that their registration procedure in MITUN be completed by the study staff. Hence, the registration of the practitioners was done by the study staff by calling the designated number. An implementation period of about 6 months was given to the private medical practitioners for notifying TB cases using MITUN. A reminder SMS for notifying TB cases was sent fortnightly. After the implementation period,a post-intervention questionnaire was administered to the practitioners. The questions were focused on the feasibility of utilizing MITUN for TB notification which included number of TB cases diagnosed/ treated during the implementation period, TB cases notified using MITUN, reasons for not using MITUN, comfort in using MITUN, opinion on scaling up and suggestions to improve TB notification using MITUN.

It was planned to recruit a minimum of 100 private medical practitioners for this study during the recruitment period of 3 months. The private practitioners were requested to continue to notify TB cases to the Nodal TB Officer as per the requirement of Govt. order through NIKSHAY after the completion of MITUN study period.

### Ethical considerations

The study was approved by the Institutional Ethics Committee of National Institute for Research in TB (NIRT) vide IEC No. 2013009. The consent forms used in the study were approved by the Institutional Ethics Committee. Written informed consent was obtained from the study participants prior to participation in the study.

### Data Analysis

The responses to the questionnaires were double verified, entered in the EpiData Version 3.1 and analysed using IBM ® SPSS® Statistics Version 20.0. Descriptive statistics of the responses to the questions was calculated.

## Results

**Fig 2:**
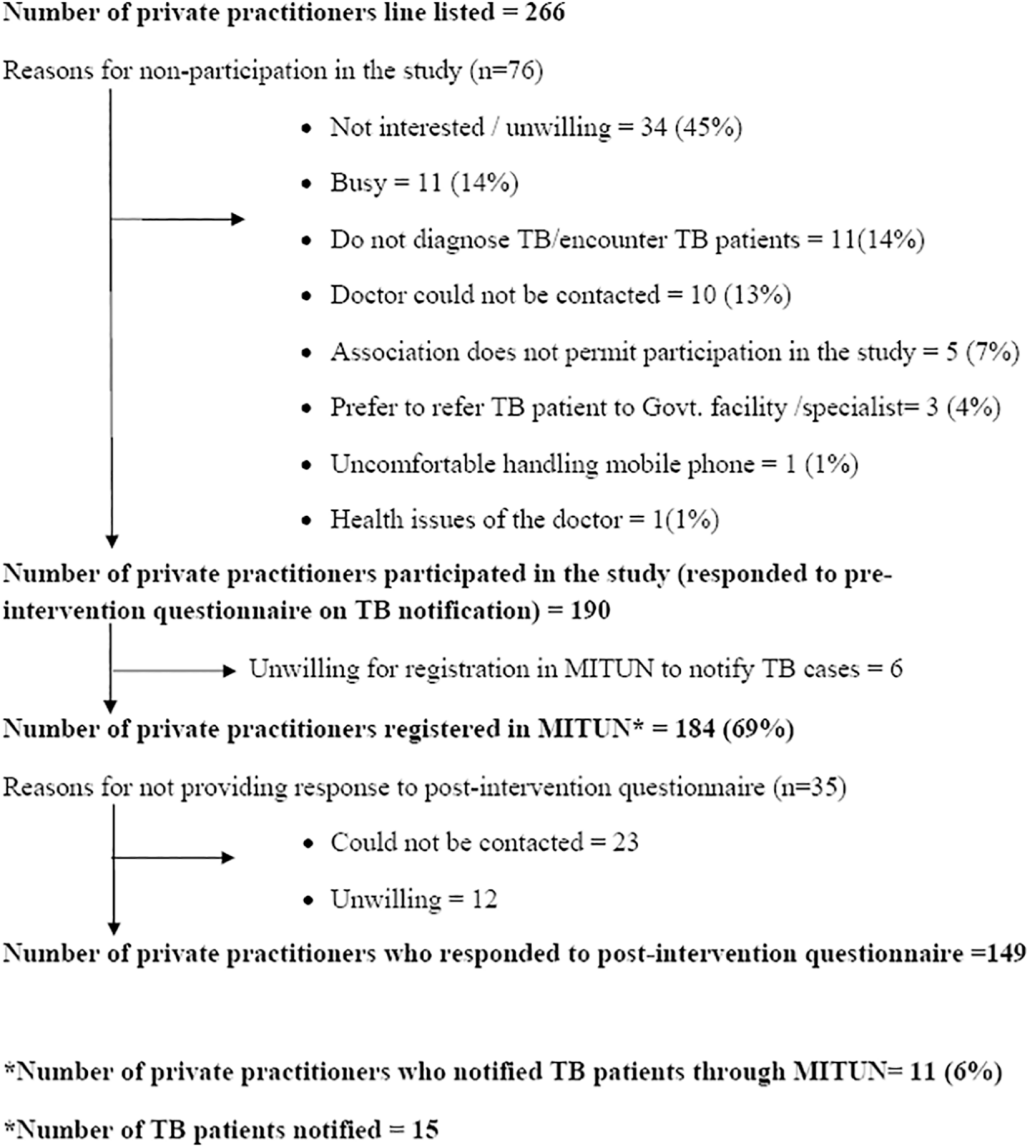
Private Medical Practitioners contacted for the study and the number of practitioners who notified TB cases through MITUN.

A total of 266 private medical practitioners were contacted for the study. Among them, 190 (71%) were willing to participate in the study and provided responses to the pre-intervention questionnaire. (Fig 2) The reasons given for non-participation in the study in the remaining 76 were primarily not interested/ unwilling (45%), busy (14%) and do not diagnose/encounter TB patients (14%). Religious Associations which did not permit disclosure of patient related details prevented participation of 5 practitioners in the study. Of the 190, there were 184 practitioners who were willing to get registered for TB notification in the MITUN project. Of the 184, 11 (6%) practitioners subsequently used MITUN for TB notification. (Fig 2)

Among the 190 practitioners who participated in the study, 148 (78%) had a post-graduate qualification; their specialisation was as follows: Chest (n = 12), ENT (n = 12), Medicine (n = 26), Pediatrics (n = 27), Surgery (n = 11), Orthopaedics (n = 11), others eg. Gynaecologist, dermatologist, radiologist, diabetologist (n = 49).

Table 2 shows the responses of the Private Medical Practitioners to the pre intervention questionnaire regarding TB notification. About 65% had diagnosed or treated TB patients in the past 6 months, 138 (73%) were aware of the Govt. Order on mandatory TB notification, 179 (94%) were comfortable in notifying TB patients to the Govt and 55(29%) had ever notified a TB case before. Mobile phone was considered a convenient modality for TB notification by 154 (81%) and by email by 46 (24%) practitioners.

**Table 2:** The responses of the Private Medical Practitioners for the pre-intervention questionnaire regarding TB notification.

	Number responded	n(%)
Number of practitioners who have diagnosed a TB patient in the past 6 months	190	124(65%)
Number of practitioners who have treated a TB patient in the past 6 months	124	80(65%)
Number of practitioners who were aware of Govt. Order on mandatory TB notification	190	138(73%)
Number of practitioners who were comfortable in notifying TB patients to the Govt.	190	179(94%)
Number of practitioners who had ever notified TB cases before	190	55(29%)
Convenient modality of notifying TB patients to the Govt.	190	
Mobile phone		154(81%)
Email		46(24%)
Post		8(4%)
Others (reference letter)		7(4%)

The feasibility of using MITUN for TB notification based on the responses from the post-intervention questionnaire obtained from 149 (81%) practitioners enrolled is shown in Table 3. There were 52 (35%) who had diagnosed at least one TB patient during the 6 month MITUN implementation period, of whom 5(10%) diagnosed more than 5 cases. A total of 155 TB cases were diagnosed of which 15 (10%) cases were notified through MITUN. Of the 52 doctors who diagnosed TB cases, 6 responded that they had notified TB patients using MITUN. Among the 6 who used MITUN for TB notification, 3 of them said that they found it user friendly while the others felt it was a time consuming, complicated process. The main reasons for not using MITUN for TB notification was due to lack of time, referral of patients to government facility for treatment, issues related to patient confidentiality and technical problems (busy phone line or call interruption).

**Table 3:** The responses of the Private Medical Practitioners for the post-intervention questionnaire regarding feasibility of using MITUN for TB notification.

	Number responded	n(%)
Number of practitioners who have diagnosed atleast one TB patient during the 6 months	149	52(35%)
Number of practitioners who notified TB case through MITUN	52	6
Reasons for not using MITUN for TB notification	45	
No time		13(29%)
Phone line was busy		8(18%)
Call was interrupted		3(7%)
Patient Confidentiality issues		8(18%)
Others (mainly patients referred to Govt. Hospitals or treatment centres after diagnosis)		16(36%)
Number of practitioners who said that MITUN can be scaled up widely to be used by private practitioners	149	57(38%)
Suggestions to improve TB notification through MITUN	137	
Complicated and long process, make it simple		29(21%)
Mobile SMS / Missed call option convenient		21(15%)
Simplify the questions with minimal crucial details		15(11%)
Meet doctors often, regular visit to clinic, create awareness		9(7%)
Make it user-friendly		65(47%)

There were 53 (38%) who felt that MITUN could be scaled up widely for use. Suggestions for improvement of MITUN were provided by 137 practitioners who mainly responded towards making the TB notification process user-friendly (47%). Simplification of the MITUN process for example reducing the call duration, including only crucial questions, using missed call or SMS option via mobile phone, making frequent visits to the clinic for meeting the doctors and creating awareness were some suggestions.

## Discussion

Interactive Voice Response System (IVRS) has been used in health care especially for treatment adherence and behavioural intervention **[10–13].** This study is one of the first of its kind to explore the utility of IVRS for TB notification. The findings from this pilot study have shown that the performance of MITUN voice based system for TB notification was sub-optimal, with 6% utilisation by a group of private medical practitioners. Nevertheless, this study has highlighted important perceptions of private providers on the intervention and provides scope for improvement.

Majority of the practitioners had responded that mobile phone was a convenient modality for notifying TB patients to the Govt. This is suggestive of a positive inclination towards using Information Communication Technology (ICT) tools to notify TB disease among private practitioners. Despite this, only 6% utilised MITUN for TB notification. Not encountering a TB case during the implementation period was a major limiting factor towards under utilisation of MITUN since only 35% of the practitioners had diagnosed or treated a TB case as per the post-intervention response. However, non-participation rate of 19% in the post-intervention questionnaire prevented us from further quantifying the TB case load of the private practitioners in the study.

The technical reasons that prevented MITUN usage were call interruption and busy phone line which inhibits the acceptance of such a system as most practitioners would not dial again if such issues occurred. Although only 11 practitioners referred to technical reasons, it is imperative that technical problems be averted. Private medical practitioners have busy schedules and their involvement in TB notification would be jeopardised if technical problems occurred.

The busy schedule of the private practitioners prevented enrolment to the study in 11(14%) and prevented TB notification through MITUN in 8. Scarcity of time is an important impediment for TB notification and this indicates the need for user friendly interventions. It also warrants repeated reminders about notification of TB cases, at regular intervals.

Confidentiality and security of information is a perceived barrier for TB notification from the point of view of private practitioners. Stigma related to TB disease further prevents information disclosure **[14]**. Patient confidentiality related issues prevented 8 practitioners from notifying TB patients. However, patient confidentiality can be breached in case of communicable and notifiable diseases, since protection of public health outweighs maintenance of patient confidentiality. According to the Medical Council of India (MCI) code of Ethics–Rules & regulations 2002, Chapter 7, Point 7.14, it is the duty of all registered medical practitioners to divulge this information to the authorized notification official as regards communicable and notifiable diseases **[15]**. It is important that private medical practitioners are made to re-visit the MCI rules to comply with TB notification procedures.

The referral of patients to Govt. Hospitals or RNTCP centres was another reason not to notify through MITUN for 16 practitioners. The underlying notion is that the patients reach the Govt. facility for treatment and in the process get automatically notified through the public health system. However, as per the Govt. order on mandatory TB notification, the notification process has to be done once a TB case is diagnosed **[3]**. Referral of TB patients to the Govt. facility for treatment once diagnosed in private sector is well appreciated; however the referral has to be done once the patients diagnosed are notified.

There were 38% who mentioned that MITUN can be scaled up widely for TB notification and suggestions were provided to improve the system. They felt that notification through MITUN was a complicated process and there was a felt need to simplify the questions. The one-time registration comprising of 4 questions takes about 2 minutes. This was done by the study staff as was preferred by the practitioners. This implies that the process of registering the practitioners into the system for notification requires assistance to achieve optimal registration. The TB notification process takes about 5 minutes and 15 questions have to be answered. The feedback from the practitioners highlight the need for limited patient related details through mobile phones.An application which is simple and less time consuming with minimum questions was considered favorable. This is further supported by the preference of SMS and missed call option by some practitioners.These findings suggest that interventions using ICT channels cannot be a direct replica of paper based methodology. Some adaptations are required for efficient functioning of ICT applications. The RNTCP has introduced the mobile App. in 2014 for TB notification from private doctors, the utility of which is yet to be studied. The extended call duration could also be attributed to the inherent difficulty with the IVRS technology due to voice input error which requires the users to repeat the responses until clarity in speech recognition is achieved. Voice recognition difficulties though not mentioned by private practitioners in our study have been documented in a previous study using IVRS **[10]**. This emphasizes the need for more effective call-flow design for better voice recognition.

The limitations of this study include a potential selection bias due to non-participation of 29% of the private practitioners approached for the study initially and 19% of them for the post-intervention responses and the lower than anticipated number of practitioners diagnosing at least one TB case (35% versus 65%) during the intervention period. These limit the external validity of the study findings. In addition there was a lack of information on the notification practices apart from MITUN in the post-intervention questionnaire.

Health surveys, in the context of Mobile Health or mHealth, are defined as the use of mobile devices for health-related data collection and reporting **[16]**. Improved accuracy, reductions in time and cost, and improved data quality are benefits of mhealth **[16]**. We attempted to use mHealth through IVRS for TB notification in our study. A previous study documented lowest error rate in the voice method, followed by electronic forms and SMS **[17]**. Voice also allows for verification to be performed easily and a voice interface can be replicated very easily in other contexts- no special software or cue cards need to be developed, and any cell phone can be used **[17]**. Hence IVRS is a promising intervention in health surveys.

A recent systematic review and meta-analysis on the Effectiveness of Mobile-Health Technologies to improve health care service delivery processes concluded that the technology has modest benefits **[18]**. This highlights the fact that mobile technology is still evolving and there is scope for improvement based on the context of its use.This pilot study has shown that the performance of MITUN voice based system for TB notification in the present format was sub-optimal. In addition, the underlying non-technical reasons that prevent TB notification from private practitioners have to be addressed for success of interventions related to TB notification. Effective strategies for enabling voluntary rather than obligatory compliance for notification of TB disease from private sector have to be considered.

## References

[1] HMSO: The Public Health (Control of Diseases) Act. Crown Copyright 1984.

[2] HMSO: The Public Health (Infectious Diseases) Regulations. Crown Copyright 1988.

[3] Notification of TB cases. Dated 7^th^ May, 2012. Government of India. Ministry of Health and Family Welfare. Available: http://www.tbcindia.nic.in/pdfs/TB%20Notification%20Govt%20%20Order%20dated%2007%2005%202012.pdf. Accessed 2015 January 2.

[4] Sachdeva KS, Satyanarayana S, Dewan PK, Nair SA, Reddy R, Kundu D, et al (2011) Source of Previous Treatment for Re-Treatment TB Cases Registered under the National TB Control Programme, India. PLoS ONE 6(7):e22061 10.1371/journal.pone.0022061 21814566 PMC3140992

[5] Wells WA, Ge CF, Patel N, Oh T, Gardiner E, Kimerling ME (2011) Size and Usage Patterns of Private TB Drug Markets in the High Burden Countries. PLoS ONE 6(5): e18964 10.1371/journal.pone.0018964 21573227 PMC3087727

[6] Revised National TB Control Programme. NIKSHAY web portal. Available: http://nikshay.gov.in/User/Login.aspx. Accessed 2015 January 2.

[7] Skinner C, Finkelstein J (2008) Review of Mobile Phone Use in Preventive Medicine and Disease Management. From Proceeding (619) Telehealth and Assistive Technologies.

[8] Ganesan M, Prashant S, Mary VP, Janakiraman N, Jhunjhunwala A, Waidyanatha N (2011) The Use of Mobile Phone as a Tool for Capturing Patient Data in Southern Rural Tamil Nadu, India. J Health Inform Dev Ctries 219–227.

[9] Jhunjhunwala A, Anandan V, Prashant S, Sachdev U (2011) Experiences with Voice Based Data Entry System over Mobile Phone in Rural India. Infrastructures for Health Care. Global Health care. Proceedings of the 3^rd^ International workshop. Page 59–66. Available: http://www.itu.dk/people/lrc/ProcInfraHealth2011.PDF Accessed 2015 September 3.

[10] Reidel K, Tamblyn R, Patel V, Huang A (2008) Pilot study of an interactive voice response system to improve medication refill compliance. BMC Med Inform Decis Mak 8:46 10.1186/1472-6947-8-46 18845004 PMC2588437

[11] Bender BG, Apter A, Bogen DK, Dickinson P, Fisher L, Wamboldt FS, et al (2010) Test of an interactive voice response intervention to improve adherence to controller medications in adults with asthma. J Am Board Fam Med 23(2):159–65. 10.3122/jabfm.2010.02.090112 20207925

[12] Regan S, Reyen M, Lockhart AC, Richards AE, Rigotti NA (2011) An interactive voice response system to continue a hospital-based smoking cessation intervention after discharge. Nicotine Tob Res 13(4):255–60. 10.1093/ntr/ntq248 21330278 PMC3107613

[13] Kaminer Y, Litt MD, Burke RH, Burleson JA (2006) An interactive voice response (IVR) system for adolescents with alcohol use disorders: a pilot study. Am J Addict 15 (Suppl 1):122–5. 17182427 10.1080/10550490601006121

[14] Courtwright A, Turner AN (2010) Tuberculosis and Stigmatization: Pathways and Interventions. Public Health Rep 125(Suppl 4): 34–42 20626191 10.1177/00333549101250S407 PMC2882973

[15] Frequently asked questions. Tuberculosis notification in India. Available: http://www.srmuniv.ac.in/downloads/FAQs_for_tb_notification_in_india.pdf. Accessed 2015 September 3.

[16] World Health Organization (2011) Global observatory for ehealth series. Volume 3. Available: http://www.who.int/goe/publications/goe_mhealth_web.pdf. Accessed 2015 January 2.

[17] Patnaik S, Brunskill E, Thies W (2009) Evaluating the accuracy of data collection on mobile phones: A study of forms, SMS, and voice. International Conference on Information and Communication Technologies and Development (ICTD): 74–84.

[18] Free C, Phillips G, Watson L, Galli L, Felix L, Edwards P, et al (2013) The Effectiveness of Mobile-Health Technologies to Improve Health Care Service Delivery Processes: A Systematic Review and Meta-Analysis. PLoS Med 10(1): e1001363 10.1371/journal.pmed.1001363 23458994 PMC3566926

